# Doctoral theses in France (1985–2025): A linked dataset of PhDs, academic networks, and institutions

**DOI:** 10.1016/j.dib.2026.112947

**Published:** 2026-06-06

**Authors:** William Aboucaya, Dastan Jasim

**Affiliations:** ACSS – DRM, Université Paris Dauphine - PSL, Pl. du Maréchal de Lattre de Tassigny, Paris 75016, France

**Keywords:** Doctoral theses, Academic networks, Bibliometrics, Gender inference, PhD jury composition

## Abstract

This paper presents a comprehensive dataset of doctoral theses defended in France between 1985 and 2025, constructed from multiple national academic metadata sources. The dataset is primarily based on the French national thesis platform and enriched with authority and bibliographic databases to improve data quality, completeness, and interoperability. The data production pipeline integrates heterogeneous sources, corrects inconsistent identifiers, enriches person and institution records, and derives variables describing academic careers, jury participation, institutional affiliations, and thesis characteristics. Additional identifiers from major academic repositories and library catalogs are integrated to facilitate linkage with external data sources and future extensions. The resulting dataset provides structured information at the thesis, individual, and institutional levels, supporting research on doctoral education, academic networks, supervision practices, jury composition, institutional collaboration, and the evolution of research communities. The paper documents the data sources, processing pipeline, feature construction, data quality issues, and limitations to facilitate reuse and future longitudinal analyses. The dataset is publicly available on Zenodo (https://doi.org/10.5281/zenodo.19453190).

Specifications Table

**Table:** 

Subject	Social Sciences
Specific subject area	French PhD theses and jury networks in France (1985–2025)
Type of data	*.csv file (dataset)* *.parquet file (dataset)* *.pdf file (appendix and codebook)*
Data collection	*Data were collected from Thèses.fr JSON and public API. 168 IdRef identifiers were manually corrected. Person- and institution-level metadata were enriched by parsing individual IdRef RDF files using rdflib and SPARQL queries. Gender was inferred using the ‘nomquamgender’ Python package. Derived variables capturing academic seniority and jury participation were computed programmatically.*
Data source location	*Institution: Agence Bibliographique de l'Enseignement Supérieur (ABES)* *City/Region: Montpellier, France* *Country: France* Primary data sources: Thèses.fr (https://www.data.gouv.fr/datasets/theses-soutenues-en-france-depuis-1985); IdRef (https://www.idref.fr); Thèses en Ligne / HAL (https://theses.hal.science); SUDOC (https://www.sudoc.fr)
Data accessibility	Repository name: Doctoral Theses in France (1985–2025) Data identification number: https://doi.org/10.5281/zenodo.19453191 Direct URL to data: https://zenodo.org/records/19453191 No login required; data are openly accessible URL to repository for data production: https://github.com/WilliamAboucaya/phd-theses-france
Related research article	A preprint of this article was published here: https://arxiv.org/abs/2604.08619

## 1 Value of the Data

• The dataset provides structured, longitudinal information on doctoral theses defended in France between 1985 and 2025, including thesis-level, person-level, and institution-level variables. It combines jury composition, supervisor information, and persistent authority identifiers, making this dataset a rare resource for research on doctoral education and academic networks.
• Researchers in scientometrics, sociology of science, and higher education can use this dataset to study supervision practices, jury composition, institutional collaboration, career trajectories, and the evolution of research disciplines over time. The dataset covers all major research fields represented in the French doctoral system.
• The dataset enables the construction of co-occurrence networks of researchers who serve on thesis defense juries. Nodes represent individuals and edges represent co-participation, supporting graph-based analyses of academic communities, sub-field boundaries, and network evolution using standard graph clustering methods. The longitudinal depth of the dataset further supports analyses of temporal institutional mobility, tracking how researchers move across institutions over the course of their careers.
• Multilingual metadata, including titles, abstracts, and keywords, available in French and English for most records, makes the dataset suitable for natural language processing tasks such as topic modeling, text classification, and cross-lingual retrieval at scale. The disciplinary classifications, furthermore, enable the study of interdisciplinary evolution in doctoral research over four decades.
• The inclusion of persistent identifiers from Thèses.fr, IdRef, TEL, and SUDOC enables direct linkage with external bibliographic catalogs, full-text repositories, and authority databases. Researchers can extend the dataset with publication records, citation data, institutional rankings, or disciplinary taxonomies without re-collecting data from source platforms.
• The dataset's structure is directly comparable to other national thesis repositories, facilitating comparative international studies of doctoral education. Finally, the relational graph structure of jury co-participation networks makes the dataset suitable for graph machine learning applications, including dynamic network modeling and link prediction.

## 2 Background

The objective of this data collection [1] was to develop a method for analyzing academic network structures. Doctoral theses and their defense committees constitute such a structured record of academic activity with a network structure, as each defense brings together supervisors and jury members. Those are connected through disciplinary, institutional, and professional ties, generating longitudinal data on supervision practices, jury composition, and researcher co-occurrence. Few national datasets make this information available in a form suitable for systematic quantitative research.

Comparable national platforms exist in Spain (Teseo) [2,3], Sweden (DiVA) [4], Portugal (RCAAP) [5], and the Czech Republic (Theses.cz) [6], but these either lack structured data on jury composition or do not integrate persistent person-level identifiers. Prior scraping efforts on Teseo did not include systematic authority control or demographic enrichment [2].

Thèses.fr, maintained by ABES [7,8], covers all doctoral theses defended in France since 1985 and includes structured information on the jury and supervisor, linked to persistent authority identifiers, making it a rare resource for quantitative research on doctoral education and academic networks. This dataset was constructed to support research on gendered jury networks and is intended as a general-purpose resource for scientometrics, sociology of science, and network analysis.

## 3 Data Description

**Fig. 1:**
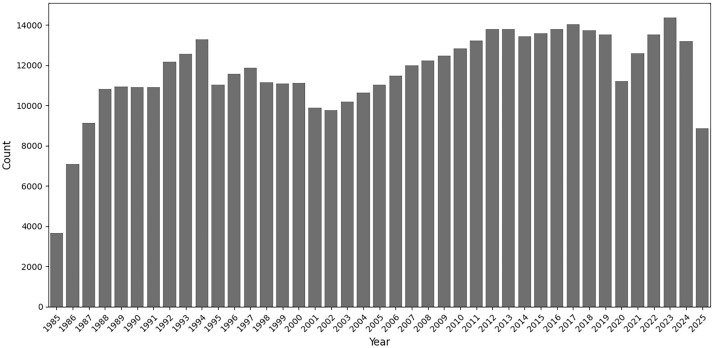
Number of theses in the dataset per year.

**Fig. 2:**
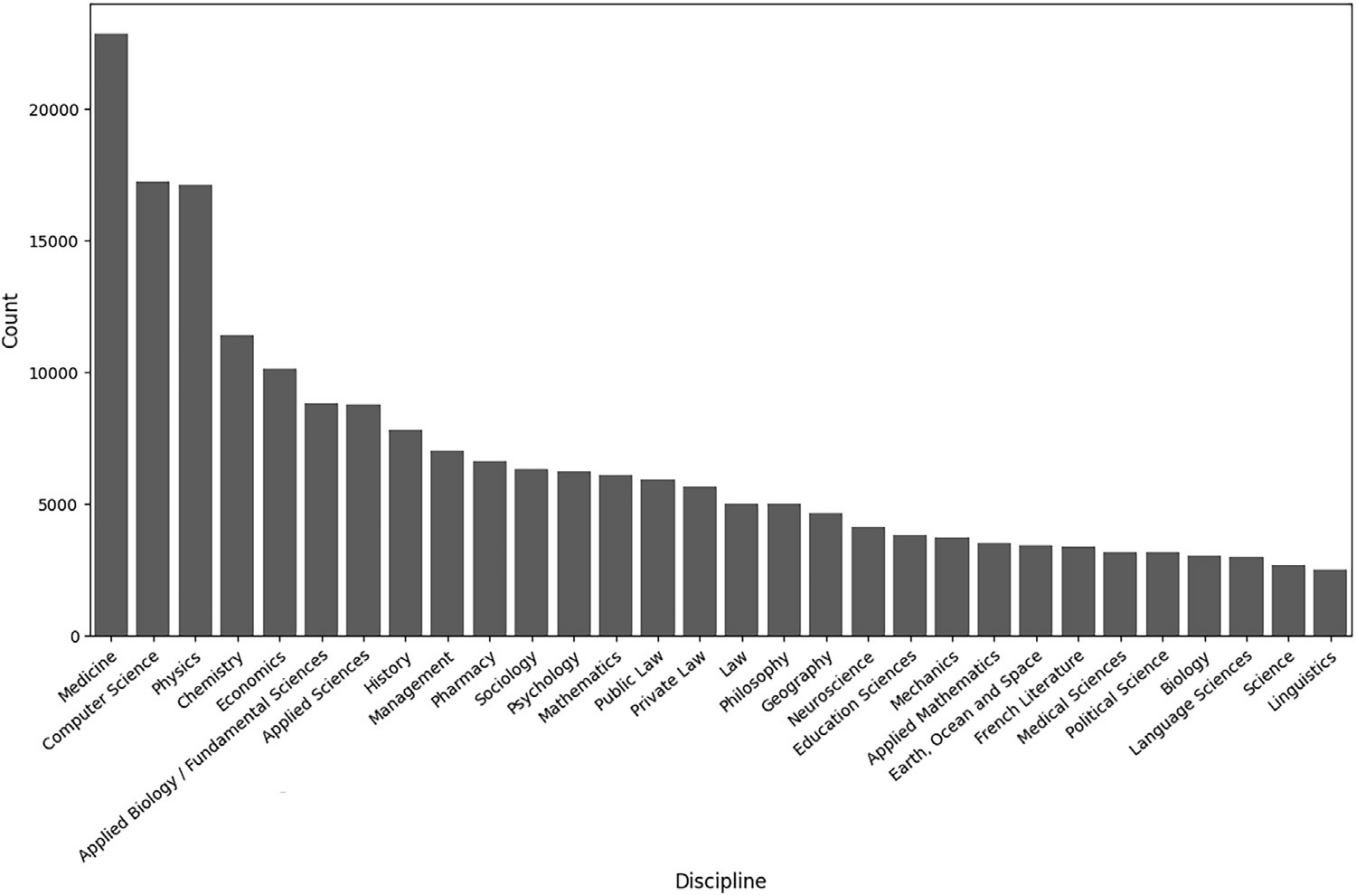
Distribution of theses by research disciplines (translated from French).

Each row of the dataset corresponds to a single Ph.D. thesis that was defended in France between 1985 and 2025 (see Fig. 1 for the year-based distribution). Overall, the dataset consists of 478,693 rows and 405 columns. A wide range of research fields is covered, including medicine, sociology, theology, and art studies (see Fig. 2). The dataset is structured to capture not only bibliographic information about the thesis itself, but also detailed information about the individuals involved in the defense, their institutional affiliations, and the thesis's content and thematic classification. For clarity and usability, the dataset's variables are organized into six main categories, described below.

• Thesis identifiers & status: This category includes unique identifiers for the thesis across different bibliographic infrastructures, as well as administrative and status-related information, such as the type of Ph.D., the defense date, and the manuscript's publication status.
• Author: This category contains information describing the doctoral candidate, including personal identifiers and demographic or biographical information when available.
• Supervisors: This category includes information about the thesis supervisor or co-supervisors, including identifiers, demographic information, and derived indicators related to academic experience and jury participation.
• Jury: This category contains information about all members of the thesis defense committee, including rapporteurs, jury members, and the jury president.
• Institutional affiliations: This category describes the research institution(s) involved in the thesis defense, the doctoral school(s), and the research partner(s) associated with the thesis.
• Content & topics: This category includes textual and thematic information describing the thesis. It contains the title, abstract, and free-text keywords in multiple languages, as well as predefined topical classifications used to categorize the thesis by discipline and research area.

**Fig. 3:**
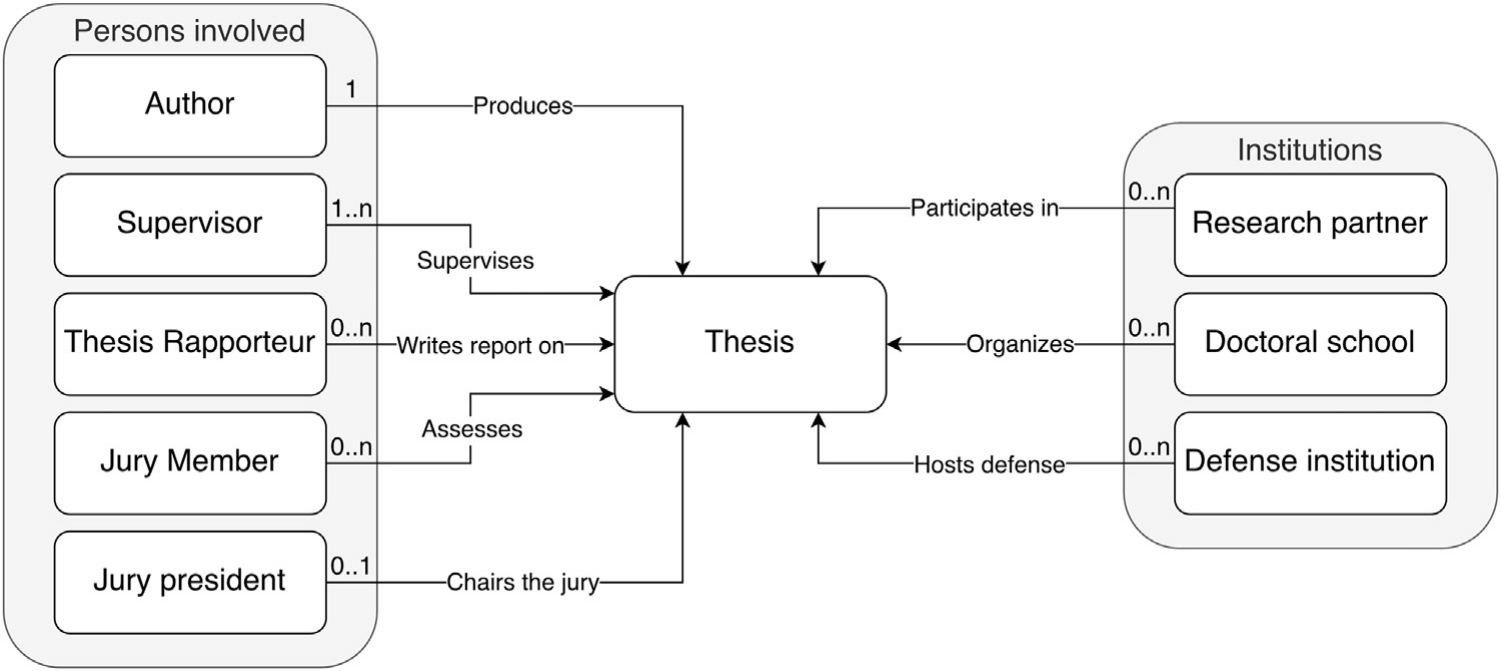
Relational logic between entities involved in a thesis.

Fig. 3 describes the involvement of the different entities associated with each thesis using quantifiers based on counts in the data, rather than on the actual number of entities which can be involved. For example, a thesis may have no jury member listed in the dataset even though it is not possible to defend a thesis without a jury. An exhaustive list of variables is provided in Table B.1 of Appendix B. In addition, Figures A.1 to A.4 of Appendix A present the proportions of missing values for selected variables in the dataset to document data completeness and potential limitations for empirical analyses. Reporting missing data is particularly crucial in this dataset as the availability of certain features varies significantly across time periods and across thesis records. In particular, Figure A.2 shows that a large proportion of theses lack detailed information about the composition of the defense jury. This is primarily due to historical data collection practices: when the Thèses.fr platform was first introduced, information about jury members was not systematically included in thesis records. As a result, older theses are less likely to contain detailed jury information, while more recent records tend to be more complete. This temporal heterogeneity in data completeness should therefore be taken into account when performing longitudinal analyses involving jury composition or participation.

**Fig. 4:**
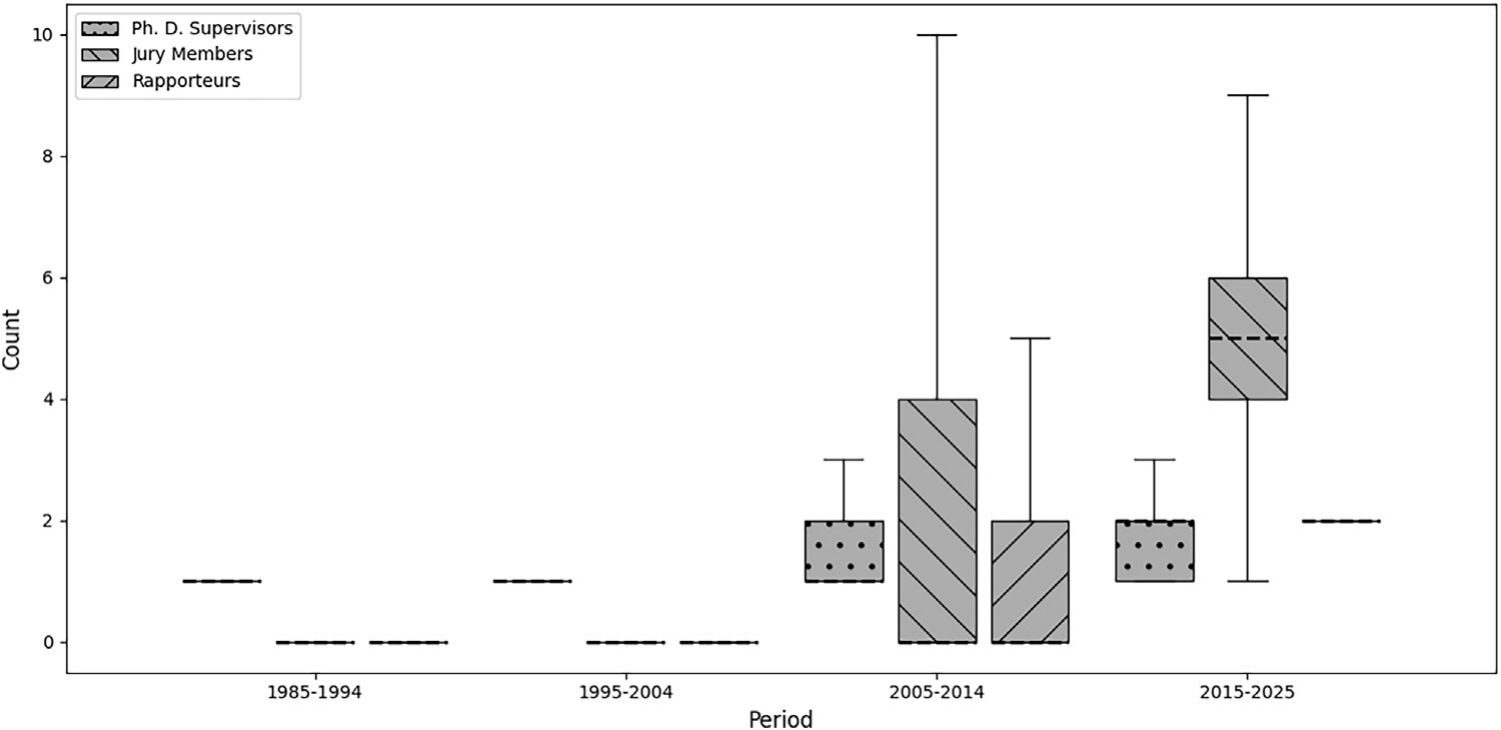
Historical development of the average number and the distribution of the number of Ph. D. supervisors, jury members, and thesis rapporteurs for each decade.

Fig. 4 further shows that the average number of supervisors, jury members, and thesis rapporteurs recorded per thesis increases over time. This trend can be explained by several factors. First, metadata recording practices have improved over time, leading to more systematic inclusion of jury-related information in thesis records. Second, jointly supervised theses have become more common, contributing to a higher number of supervisors per thesis. Third, changes in doctoral regulations regarding thesis jury composition have led to larger, more diverse juries [9].

The case of doctoral schools is similar. Doctoral schools are organizational entities within universities that oversee, for example, doctoral training, administrative procedures, and aspects of doctoral supervision and evaluation. However, these structures were not systematically established in French universities before being formally institutionalized at the national level by regulatory reforms implemented in 2006 [10]. Prior to this reform, experimental structures performing similar functions existed, but only at some universities or in some regions. As a result, information related to doctoral schools is almost systematically absent from thesis records before 1990 and remains very sparse between 1990 and 2006. This absence should therefore not be interpreted as missing data in the usual sense, but rather as the consequence of the historical evolution of doctoral education institutions in France. In the data entries after 2006, the presence of doctoral school information in thesis records becomes much more systematic, reflecting the institutionalization of doctoral schools.

It should be noted that the names of doctoral schools and other academic institutions may change over time as a result of mergers, reorganizations, or renaming processes. The dataset preserves the institution names as they appear in the original thesis records, meaning that the same institution may be represented under different names across time periods. Consequently, for longitudinal analyses or institution-level aggregation, IdRef identifiers should be preferred, as they provide a more stable and reliable representation of institutional identity.

Certain features, for example, those describing a thesis jury member, are made available multiple times because they describe an entity that may appear more than once in a given thesis. These versions are differentiated by a numerical index appended to the variable name (e.g., title.0, jury_member.1, etc.), allowing access to information about each individual version of the entity. For each indexed feature, the index values are inherited directly from the original Thèses.fr dataset. To the best of our knowledge, this order is arbitrary and does not reflect an explicit hierarchy in the thesis record. For example, language.0 is not necessarily the primary language of the thesis manuscript. The maximum cardinality of each feature was derived empirically from the dataset and corresponds to the largest number of occurrences of that feature observed in a single thesis record. For each group of columns associated with a person (author, supervisor, rapporteur, jury member, or jury president), the dataset includes their IdRef identifier, first name, last name, gender (when available or inferred), country, birth and death dates, as well as information related to their languages of writing, when available.

Another feature-naming pattern concerns the variables describing the thesis's textual content, namely its title, abstract, and free-form topic keywords. Because these textual elements may be available in multiple languages for a single thesis, multiple versions of each feature are included in the dataset. For each of these indexed features, an additional column with the same base name and the suffix .language is provided (e.g., title.0.language). This companion variable specifies the language associated with the corresponding textual field, allowing users to identify and filter content by language. This structure allows preservation of all available multilingual information while maintaining a tabular dataset format. Although most theses in the dataset are written primarily in French (∼ 88%), a large proportion of theses also include an English version of their titles (∼ 81%) and abstracts (∼ 59%). The distribution of languages across all textual variables is presented in Table A.1.

## 4 Experimental Design, Materials and Methods

Table 1 shows the workflow of the data creation. The dataset is primarily derived from a main source, Thèses.fr, the official French national platform for the dissemination of doctoral theses, maintained by the Agence Bibliographique de l’Enseignement Supérieur (Higher Education Bibliographic Agency, ABES) [7]. Data processing was performed in Python (≥ 3.12) using the following packages: pandas 25.0, numpy 0.9.42, rdflib 27.0.2, nomquamgender 3.9.2. These are the ones we consider most susceptible to version changes relevant to the creation of the dataset. The full list of packages used throughout the project is available on GitHub. Thèses.fr provides comprehensive coverage of doctoral research in France by indexing all Ph.D. theses defended since 1985, as well as doctoral theses currently in preparation when they have been formally reported by academic institutions. In addition to core thesis records, the platform provides structured information on the individuals involved in each thesis, like doctoral candidates, supervisors, and jury members, as well as on the associated institutions. Each record typically includes rich bibliographic metadata such as titles, abstracts, keywords, languages, and disciplinary classifications – designed to facilitate search, retrieval, and downstream analysis. Data from Thèses.fr is made openly available through a public API and can also be downloaded as bulk datasets in CSV, JSON, or NDJSON formats [7]. The data are made available under the Etalab Open License, the open-data license adopted by most French public institutions. To construct the dataset, we relied on the JSON bulk export, as this format provides more complete data than what can be retrieved through API queries or other export formats.

**Table 1:** Overview of the data acquisition, enrichment, and export pipeline.

However, this export was published on January 8, 2024. Consequently, all theses defended after this date, as well as theses that had already been defended but had not yet been reported to ABES at the time of the dataset publication, were not included in the bulk export. To mitigate this limitation and improve temporal coverage, we complemented the dataset by retrieving missing thesis records for the years 2022 to 2025 using the public API. This complementary data-collection step was conducted on March 31, 2026. As a result, the dataset used in this study reflects the state of the Thèses.fr database as of that date. Users of the dataset should therefore be aware that theses reported to ABES after this date are not included and that future updates of the dataset may further improve coverage for the most recent years. Data from Thèses.fr are initially harvested as JSON objects, which do not impose a predefined ordering of fields. We therefore define a column ordering explicitly during the transformation of the data into a tabular format to ensure consistency across versions of the dataset.

To complement and enrich this information, we additionally rely on IdRef (Identifiants et Référentiels), the French national authority database for higher education and research, also maintained by ABES [11]. IdRef provides persistent identifiers and authority records for entities involved in scholarly production, including persons like researchers, authors, supervisors, organizations like universities and research institutions, and conferences. These authority records consolidate variant names, affiliations, and contextual information with the goal of enabling entity disambiguation and ensuring consistency across bibliographic data sources. Thèses.fr includes IdRef identifiers for most individuals and institutions referenced in thesis records, which allows for reliable linkage and cross-referencing between theses, persons, and organizations. IdRef data are openly accessible via a public API, downloadable in structured formats such as RDF and JSON, and can also be queried through a SPARQL endpoint [11], enabling advanced semantic queries and data integration. Similar to Thèses.fr, IdRef data are also distributed under the Etalab Open License.

After downloading the raw data from the source platforms, we apply a series of data enhancement steps aimed at improving overall data quality prior to feature engineering and analysis. These enhancements focus on correcting inconsistencies and omissions in the original sources that would otherwise limit interoperability, enrichment, or downstream analytical validity.

The first enhancement step concerns validating and correcting the IdRef identifiers in the Thèses.fr dataset. We observe that certain IdRef references are ill-formed or do not correspond to any existing IdRef authority record, preventing reliable linkage with external authority data. These invalid identifiers were individually reviewed and corrected by one of the authors of this paper, based on contextual information available in the thesis records and cross-referencing with the IdRef database. To ensure that no false information is introduced, only the cases where a valid IdRef record could be identified unambiguously were corrected. The full list of corrections is available in our GitHub repository. After this step, 168 IdRef identifiers have been corrected and refer to the correct record. This manual correction step enables the subsequent enrichment of person- and institution-level metadata using authoritative identifiers.

A second enhancement step addresses the incomplete coverage of gender information in IdRef authority records for persons. As gender is not systematically recorded in IdRef, many individual records lack this attribute. To mitigate this limitation, we apply an automated gender inference tool that assigns a probable gender based on the first name when sufficient confidence can be established. We have chosen to use the nomquamgender [12]. Python package which proposes state-of-the-art level performances for gender inference from first name and has already been evaluated in the context of the Spanish doctoral theses platform TESEO [13]. We parameterize the gender classifier so that it produces a label only if it has a confidence score over 80% for a gender. This process allowed us to improve the completion of our gender data from 433,340 persons with unknown gender to 37,621 after gender guessing. Note should be taken that this gender completion step, as well as the initial gender harvesting from IdRef, only produce binary gender categorization (“male” or “female”).

After those correction steps, we enrich the initial Thèses.fr dataset with data from IdRef on the persons and institutions involved in theses. First, we found that in many instances, the first and last names of persons were switched in the Thèses.fr. Therefore, we decided to keep the names from the IdRef instead, as they are typically more reliable. Additionally, for each person in a thesis record, we enriched their data with the following information from IdRef when they were available:

• Gender
• Birth date
• Death date
• Language(s) of writing
• Country

Once person-level records had been enriched and validated, we proceeded to construct additional derived features designed to facilitate quantitative and longitudinal analyses. These features were computed at both the individual and thesis levels and primarily aim to capture aspects of academic seniority and participation intensity that are not directly available in the raw sources.

At the individual level, we compute age at the time of the thesis defense for all persons involved in a given thesis, provided that their date of birth is available in the corresponding IdRef authority record. This feature enables comparative analyses of academic trajectories and generational patterns across roles like doctoral candidates, supervisors, jury members.

For Ph.D. supervisors and jury members specifically, we also derive additional role-related indicators intended to approximate academic experience and involvement in doctoral evaluation processes:

• The number of occurrences in juries during the four years preceding the thesis defense, computed dynamically with respect to each defense date, which we call centrality. This variable captures recent participation intensity in doctoral evaluation. The four-year time window is motivated by the typical duration of doctoral studies in France, which generally is between 36 and 52 months [14]. Restricting the measure to this period ensures that the indicator reflects evaluation activity occurring within a timeframe directly relevant to the preparation and supervision of the thesis under consideration. As each person occupies at most one position per thesis by construction of the dataset schema, no deduplication is applied when computing this indicator.
• The number of years since the individual defended their own Ph.D. thesis, when this information is available, is used as an approximation for academic seniority.
• The number of years since the individual first participated in a doctoral jury (either as a jury member or as a supervisor), providing an estimate of experience in doctoral supervision and evaluation.

Finally, we introduce thesis-level features that describe the linguistic and organizational characteristics of each defense. We compute the number of distinct languages in which a title or abstract is provided, which serves as an indicator of the intended international dissemination scope. In addition, we also include the total number of Ph.D. supervisors, jury members, reviewers, and distinct academic institutions associated with each thesis.

Together, these engineered features substantially extend the dataset's analytical potential beyond its original descriptive metadata, enabling more refined investigations of academic careers and institutional dynamics in doctoral education.

To further facilitate the reuse of our dataset and enable its extension through additional external data sources, we also integrate two complementary identifiers for each thesis record. These identifiers correspond to those used by Thèses En Ligne (Online Theses, TEL) [15] and the Système Universitaire de Documentation (University Documentation System, SUDOC) [16], two major infrastructures providing metadata for theses and academic documents in France. By including these identifiers, we allow our dataset to be easily linked with other repositories that contain complementary bibliographic, institutional, or full-text information.

## Limitations

Three limitations of the dataset merit acknowledgment. First, automated gender inference from first names is inherently imperfect and has proven so also in the nomquamgender package: by estimating a global cultural consensus across 36 reference sources [12] the method cannot recover naming conventions that are absent from those sources altogether like names from non-state or minority linguistic communities in particular may be misclassified or left unresolved, introducing a degree of noise into any gender-based analyses.^1^ Second, coverage for the most recent years is incomplete: theses defended in 2023, 2024, and 2025 that had not been registered on theses.fr by 31 March 2026 are absent from the dataset, which means totals for these years underrepresent actual doctoral output. Third, a small number of records predate 1985. These have been retained for completeness, as excluding them would involve an arbitrary editorial decision, but they constitute clear outliers and are excluded from time-series visualizations where their presence would distort the visual range and reduce interpretability.

## Ethics Statement

The authors have read and comply with the ethical requirements for publication in Data in Brief. This work does not involve human subjects, animal experiments, or data collected from social media platforms. The dataset is derived exclusively from publicly available administrative and bibliographic metadata sources.

## CRediT Author Statement

**William Aboucaya:** Conceptualization, Software, Validation, Formal analysis, Data Curation, Writing – Original Draft, Writing – Review & Editing. **Dastan Jasim:** Conceptualization, Software, Formal analysis, Data Curation, Writing – Original Draft, Writing – Review & Editing, Visualization.

## Declaration of Competing Interest

The authors declare that they have no known competing financiaxl interests or personal relationships that could have appeared to influence the work reported in this paper.

## Data Availability

ZenodoDoctoral Theses in France (1985–2025) (Original data)

## Appendix Supplementary materials

Image, application 1

Image, application 2

## Footnotes

1 For example, the first name Dastan, borne by one of the authors who is female, was submitted to the model and returned as strongly male, despite being unisex in Kurdish onomastic tradition, a misclassification likely traceable to the underrepresentation of Kurdish-specific records among the package's reference sources.
