# Supplementary material for: Doctoral theses in France (1985–2025): A linked dataset of PhDs, academic networks, and institutions
Source: Data Brief. 2026 Jun 6;67:112947. doi: 10.1016/j.dib.2026.112947 (PMC13276782; doi:10.1016/j.dib.2026.112947)
Supplement: Supplementary file 1 [file mmc1.docx]

**APPENDIX A**

**Percentages of missing data for selected features**

Figure A.1: Data on theses missing


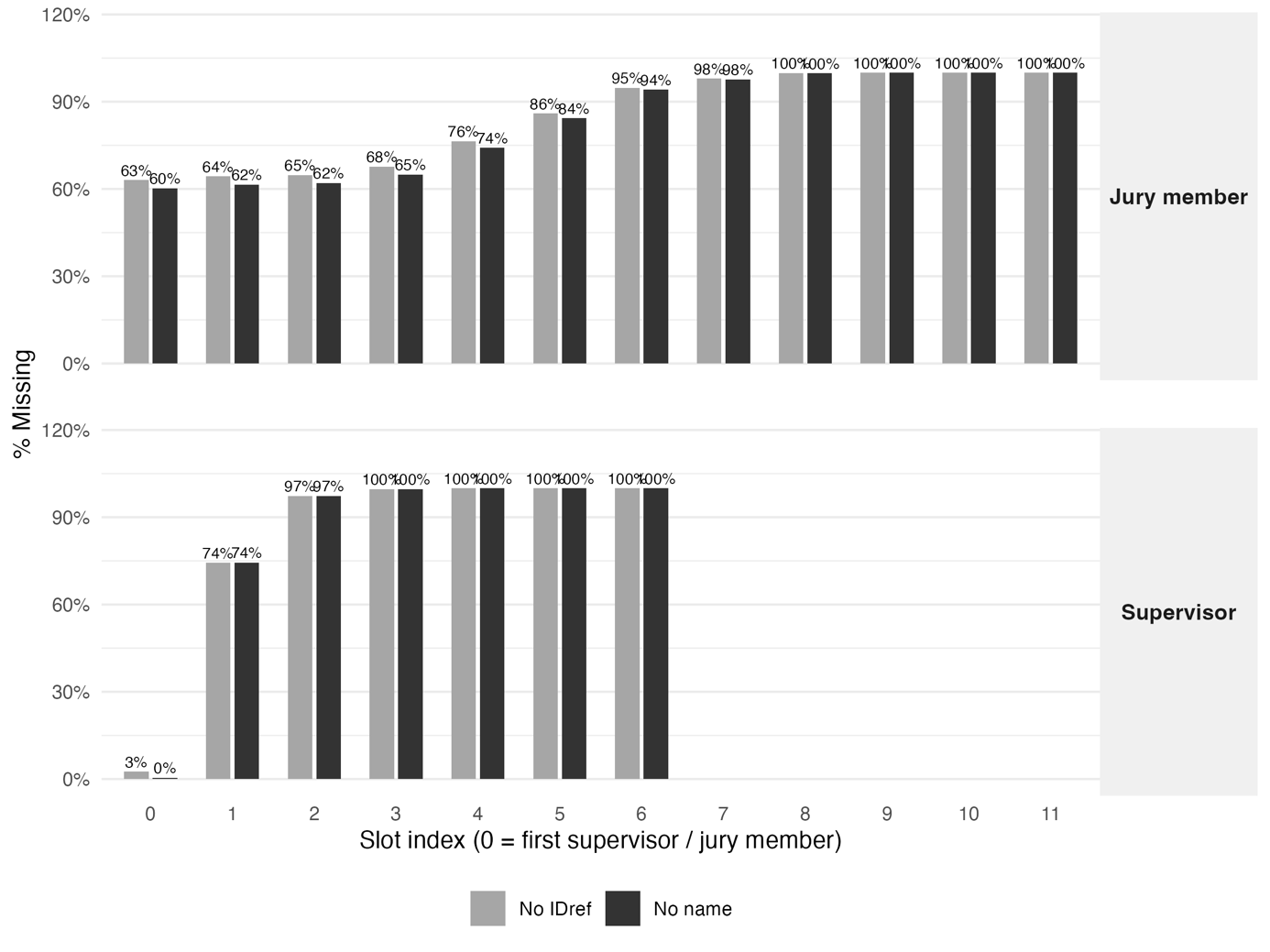


Figure A. 2: Data on supervisors and jury members missing


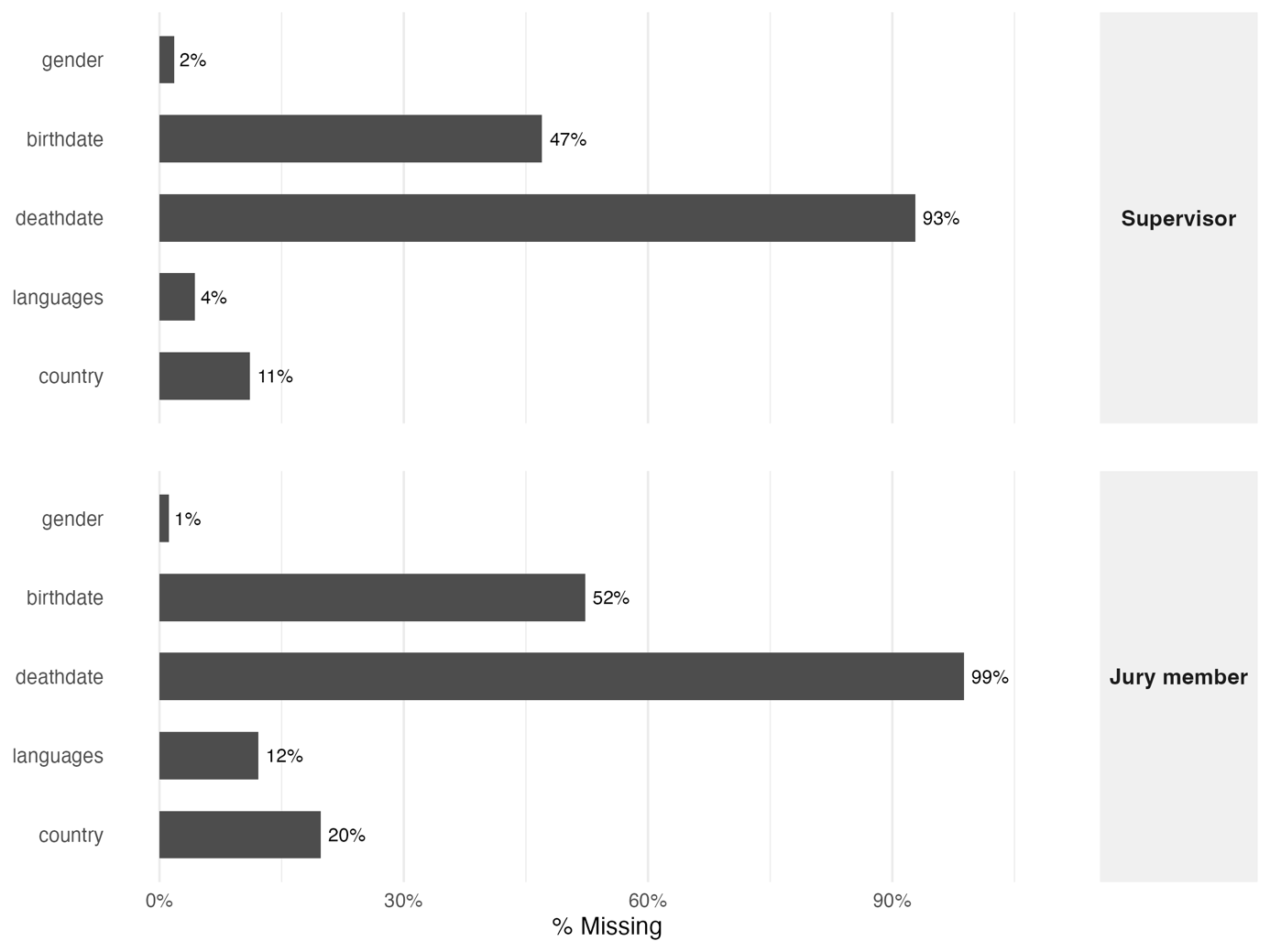


Figure A. 3: Enriched data from IdRef missing


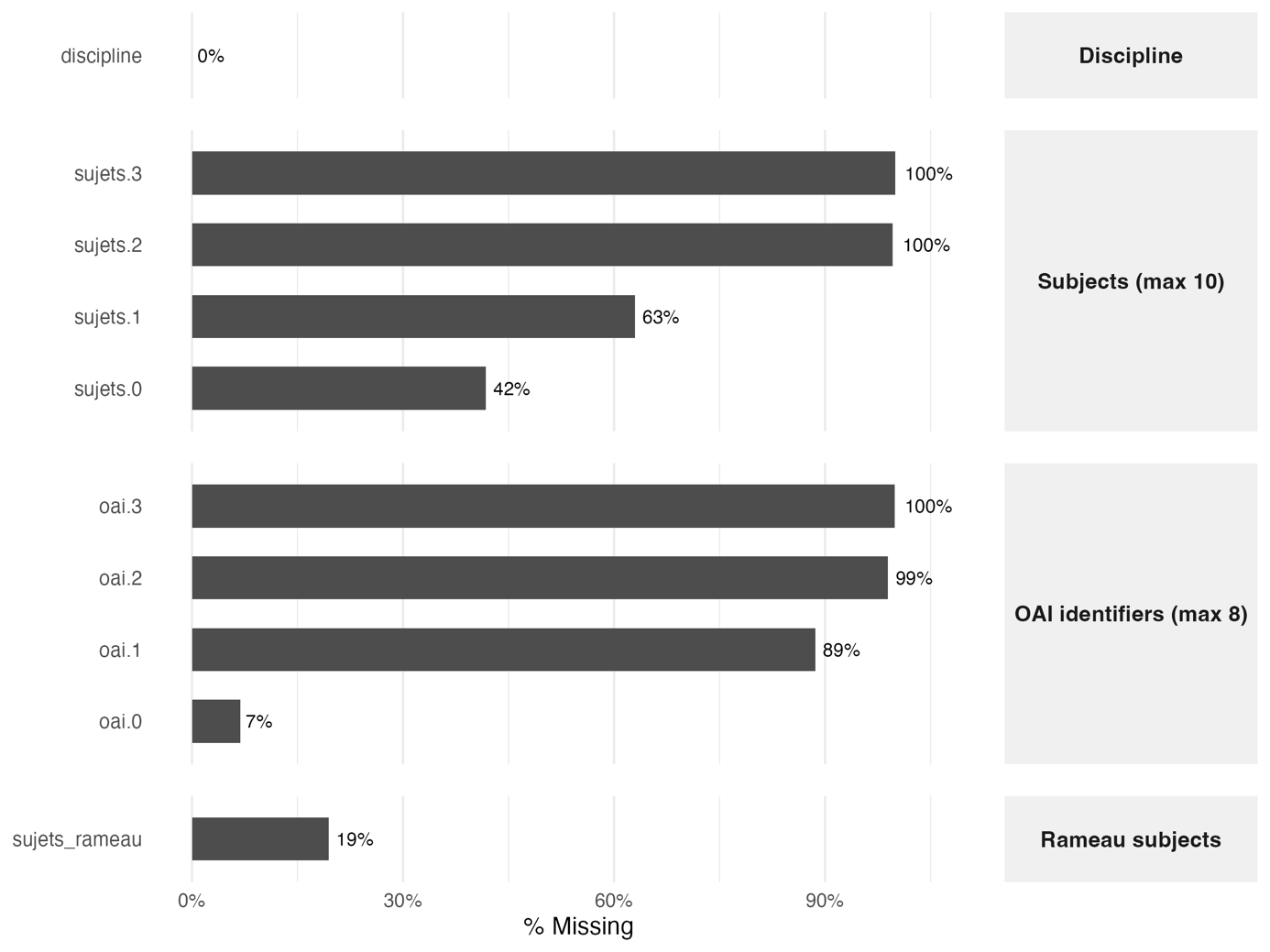


Figure A. 4: Subject entries missing


**Distribution of languages**

| **Language** | **Thesis language (0% NA)** | **Abstract (12.8% NA)** | **Title (0% NA)** | **Subject (41.7% NA)** | **Author (14.0% NA)** | **Supervisor (3.7% NA)** | **Jury (61.3% NA)** |
| --- | --- | --- | --- | --- | --- | --- | --- |
| French | 414554 (86.601%) | 416536 (87.015%) | 471600 (98.518%) | 278841 (58.25%) | 357350 (74.651%) | 454423 (94.93%) | 184341 (38.509%) |
| English | 83597 (17.464%) | 295530 (61.737%) | 392949 (82.088%) | 177345 (37.048%) | 66364 (13.864%) | 52955 (11.062%) | 82376 (17.209%) |
| Italian | 1327 (0.277%) | 428 (0.089%) | 1422 (0.297%) | 284 (0.059%) | 2937 (0.614%) | 5047 (1.054%) | 11123 (2.324%) |
| Spanish | 939 (0.196%) | 911 (0.19%) | 1346 (0.281%) | 662 (0.138%) | 3223 (0.673%) | 3674 (0.768%) | 7363 (1.538%) |
| Portuguese | 597 (0.125%) | 534 (0.112%) | 804 (0.168%) | 417 (0.087%) | 1727 (0.361%) | 1846 (0.386%) | 3130 (0.654%) |
| German | 421 (0.088%) | 263 (0.055%) | 541 (0.113%) | 168 (0.035%) | 1232 (0.257%) | 3627 (0.758%) | 8517 (1.779%) |
| Chinese | 164 (0.034%) | 66 (0.014%) | 128 (0.027%) | 47 (0.01%) | 1543 (0.322%) | 965 (0.202%) | 1404 (0.293%) |
| Arabic | 134 (0.028%) | 81 (0.017%) | 147 (0.031%) | 64 (0.013%) | 2510 (0.524%) | 1774 (0.371%) | 3121 (0.652%) |
| Russian | 97 (0.02%) | 20 (0.004%) | 81 (0.017%) | 15 (0.003%) | 426 (0.089%) | 730 (0.152%) | 1202 (0.251%) |
| Romanian | 85 (0.018%) | 18 (0.004%) | 93 (0.019%) | 12 (0.003%) | 495 (0.103%) | 827 (0.173%) | 1148 (0.24%) |
| Latin | 81 (0.017%) | - | 57 (0.012%) | 2 (0.0%) | 65 (0.014%) | 251 (0.052%) | 254 (0.053%) |
| Polish | 52 (0.011%) | 23 (0.005%) | 76 (0.016%) | 15 (0.003%) | 258 (0.054%) | 427 (0.089%) | 708 (0.148%) |
| Greek | 38 (0.008%) | 16 (0.003%) | 41 (0.009%) | 11 (0.002%) | 436 (0.091%) | 396 (0.083%) | 723 (0.151%) |
| Czech | 31 (0.006%) | 23 (0.005%) | 44 (0.009%) | 12 (0.003%) | 139 (0.029%) | 206 (0.043%) | 349 (0.073%) |
| Catalan | 29 (0.006%) | 16 (0.003%) | 34 (0.007%) | 10 (0.002%) | 51 (0.011%) | 145 (0.03%) | 356 (0.074%) |
| Japanese | 27 (0.006%) | 14 (0.003%) | 38 (0.008%) | 16 (0.003%) | 269 (0.056%) | 178 (0.037%) | 503 (0.105%) |
| Hungarian | 25 (0.005%) | 10 (0.002%) | 23 (0.005%) | 1 (0.0%) | 101 (0.021%) | 195 (0.041%) | 259 (0.054%) |
| Hebrew | 19 (0.004%) | 1 (0.0%) | 16 (0.003%) | 1 (0.0%) | 48 (0.01%) | 143 (0.03%) | 219 (0.046%) |
| Breton | 17 (0.004%) | 4 (0.001%) | 16 (0.003%) | 2 (0.0%) | 47 (0.01%) | 68 (0.014%) | 56 (0.012%) |
| Vietnamese | 17 (0.004%) | 21 (0.004%) | 24 (0.005%) | 16 (0.003%) | 450 (0.094%) | 228 (0.048%) | 290 (0.061%) |
| Basque | 16 (0.003%) | 11 (0.002%) | 24 (0.005%) | 9 (0.002%) | 29 (0.006%) | 38 (0.008%) | 59 (0.012%) |
| Bulgarian | 11 (0.002%) | 4 (0.001%) | 13 (0.003%) | 4 (0.001%) | 73 (0.015%) | 139 (0.029%) | 135 (0.028%) |
| Persian | 10 (0.002%) | 7 (0.001%) | 8 (0.002%) | 6 (0.001%) | 140 (0.029%) | 112 (0.023%) | 257 (0.054%) |
| Ukrainian | 10 (0.002%) | 4 (0.001%) | 7 (0.001%) | 2 (0.0%) | 75 (0.016%) | 50 (0.01%) | 76 (0.016%) |

Table A. 1: Language distribution across thesis metadata fields. Column headers show percentage of theses with no language info recorded
