# Supplementary material for: Doctoral theses in France (1985–2025): A linked dataset of PhDs, academic networks, and institutions
Source: Data Brief. 2026 Jun 6;67:112947. doi: 10.1016/j.dib.2026.112947 (PMC13276782; doi:10.1016/j.dib.2026.112947)
Supplement: Supplementary file 2 [file mmc2.docx]

**APPENDIX B**

**Variables codebook**

Table B. 1: Overview of variable groups in the dataset

| **Group** | **Type** | **N slots** | **Variables** | **Description** |  |
| --- | --- | --- | --- | --- | --- |
| ***Thesis identifiers & status*** | | | | | |
|  | string | 1 | nnt | National thesis number (unique identifier) |  |
|  | string | 1 | tel | Thèses en ligne thesis number (unique identifier) |  |
|  | string | 1 | ppn | SUDOC Pica Production Number (unique identifier) |  |
|  | boolean | 1 | accessible | Whether the thesis is publicly accessible (true) or not (false) |  |
|  | datetime | 1 | embargo | Embargo date if applicable. Not available for theses gathered using the API. |  |
|  | string | 1 | cas | System used to publish the thesis record |  |
|  | string | 1 | source | Data source |  |
|  | boolean | 1 | phd_by_publication | true if the doctorate is a thesis by published works, false otherwise. Not available for theses gathered using the API. |  |
|  | string | 1 | code_etab | Institution code |  |
|  | datetime | 1 | defense_date | Thesis defense date |  |
|  | boolean | 1 | from_api | true if the thesis has been gathered from the Thèses.fr API, false if it is from the 2023 bulk export |  |
| ***Author*** | | | | | |
|  | string | 1 | author.idref | IdRef authority identifier |  |
|  | string | 1 | author.lastname, author.firstname | Last and first name |  |
|  | string | 1 | author.gender | Gender |  |
|  | datetime | 1 | author.birthdate, author.deathdate | Birth and death date |  |
|  | string | 1 | author.languages | Languages spoken |  |
|  | string | 1 | author.country | Country of origin |  |
| ***Supervisors*** | | | | | |
|  | string | 0–6 | supervisor.{i}.idref | IdRef authority identifier |  |
|  | string | 0–6 | supervisor.{i}.lastname, supervisor.{i}.firstname | Last and first name |  |
|  | string | 0–6 | supervisor.{i}.gender | Gender |  |
|  | datetime | 0–6 | supervisor.{i}.birthdate, supervisor.{i}.deathdate | Birth and death date |  |
|  | string | 0–6 | supervisor.{i}.languages | Languages spoken |  |
|  | string | 0–6 | supervisor.{i}.country | Country of origin |  |
|  | number | 0–6 | supervisor.{i}.centrality | Number of occurrences in juries in the past 4 years |  |
|  | number | 0–6 | supervisor.{i}.age | Age at time of the thesis defense |  |
|  | number | 0–6 | supervisor.{i}.yrs_since_phd | Years since the person's thesis defense |  |
|  | number | 0–6 | supervisor.{i}.yrs_since_first_jury | Years since the person's first participation in a thesis jury in France |  |
|  | number | 1 | num_supervisors | Number of supervisors |  |
| ***Jury*** | | | | | |
|  | string | 0–11 | jury_member.{i}.idref | IdRef authority identifier |  |
|  | string | 0–11 | jury_member.{i}.lastname, jury_member.{i}.firstname | Last and first name |  |
|  | string | 0–11 | jury_member.{i}.gender | Gender |  |
|  | datetime | 0–11 | jury_member.{i}.birthdate, jury_member.{i}.deathdate | Birth and death date |  |
|  | string | 0–11 | jury_member.{i}.languages | Languages spoken |  |
|  | string | 0–11 | jury_member.{i}.country | Country of origin |  |
|  | number | 0–11 | jury_member.{i}.centrality | Number of occurrences in juries in the past 4 years |  |
|  | number | 0–11 | jury_member.{i}.age | Age at time of the thesis defense |  |
|  | number | 0–11 | jury_member.{i}.yrs_since_phd | Years since the person's thesis defense |  |
|  | number | 0–11 | jury_member.{i}.yrs_since_first_jury | Years since the person's first participation in a thesis jury in France |  |
|  | number | 1 | num_jury_members | Number of jury members |  |
|  | string | 1 | jury_president.idref | Jury president IdRef identifier |  |
|  | string | 1 | jury_president.lastname, jury_president.firstname | Jury president name |  |
|  | string | 1 | jury_president.gender | Jury president gender |  |
|  | datetime | 1 | jury_president.birthdate, jury_president.deathdate | Jury president birth and death date |  |
|  | string | 1 | jury_president.languages | Jury president languages |  |
|  | string | 1 | jury_president.country | Jury president country |  |
|  | string | 0–5 | rapporteur.{i}.idref | Rapporteur IdRef identifier |  |
|  | string | 0–5 | rapporteur.{i}.lastname, rapporteur.{i}.firstname | Rapporteur name |  |
|  | string | 0–5 | rapporteur.{i}.gender | Rapporteur gender |  |
|  | datetime | 0–5 | rapporteur.{i}.birthdate, rapporteur.{i}.deathdate | Rapporteur birth and death date |  |
|  | string | 0–5 | rapporteur.{i}.languages | Rapporteur languages |  |
|  | string | 0–5 | rapporteur.{i}.country | Rapporteur country |  |
|  | number | 1 | num_rapporteur | Number of thesis rapporteurs |  |
| ***Institutional affiliations*** | | | | | |
|  | string | 0–4 | defense_institution.{i}.idref | Defense institution IdRef identifier |  |
|  | string | 0–4 | defense_institution.{i}.name | Defense institution name |  |
|  | string | 0–1 | doctoral_school.{i}.idref | Doctoral school IdRef identifier |  |
|  | string | 0–1 | doctoral_school.{i}.name | Doctoral school name |  |
|  | string | 0–7 | research_partner.{i}.idref | Research partner IdRef identifier |  |
|  | string | 0–7 | research_partner.{i}.name | Research partner name |  |
|  | string | 0–7 | research_partner.{i}.type | Research partner type |  |
|  | number | 1 | num_research_partners | Number of research partners |  |
| ***Content & topics*** | | | | | |
|  | string | 0–5 | title.{i}, title.{i}.language | Thesis title and its language |  |
|  | string | 0–7 | abstract.{i}, abstract.{i}.language | Abstract and its language |  |
|  | string | 0–9 | topics.{i}, topics.{i}.language | Keywords and their language |  |
|  | string | 1 | discipline | Discipline field |  |
|  | string | 1 | rameau_topics | Rameau topics headings (separated with \|\|) |  |
|  | string | 0–3 | language.{i} | Thesis language(s) |  |
|  | string | 0–7 | oai.{i} | Dewey Decimal Classes. Not available for theses gathered using the API. |  |
